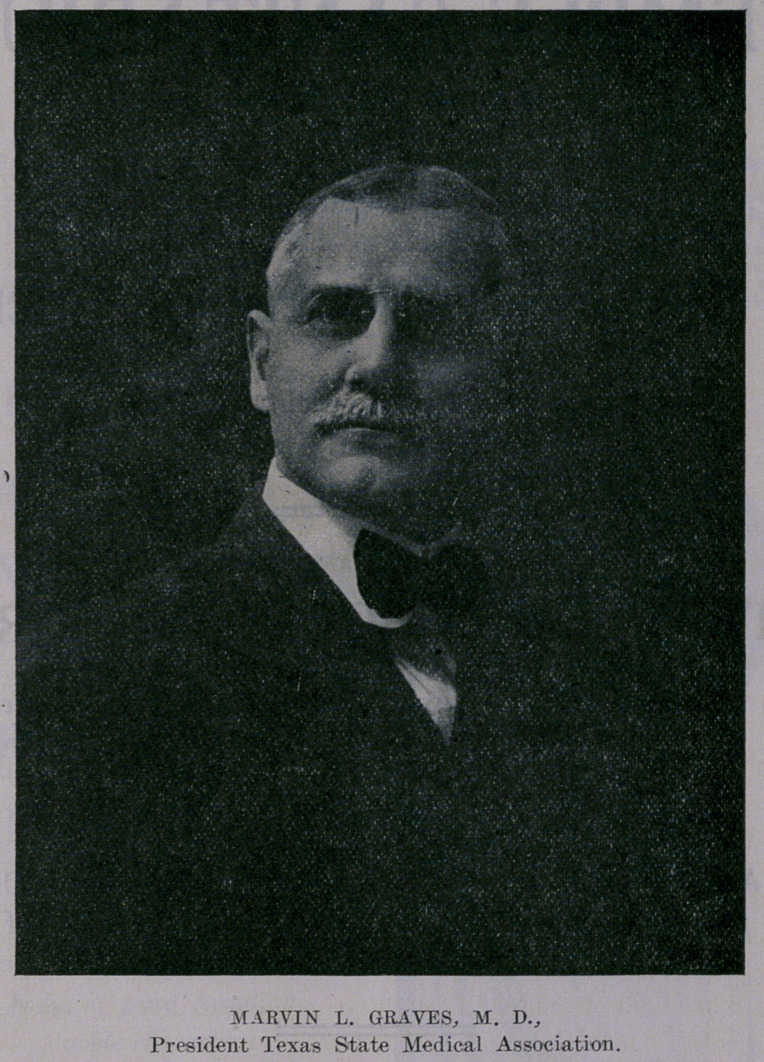# The Supreme Ideal in the Boys Education

**Published:** 1913-06

**Authors:** 


### The Supreme Ideal in the Boys Education.

When the general opinion prevails that a young man's company is not acceptable to the young lady whom he escorts for the evening unless he, at least, bestows caresses upon her; and that the young lady's company is not acceptable to the young man unless she permits of such liberties, is it a little wonder that there exists in the minds of the young people a doubt as how to regard each other in respect to their ideals of virtue and social propriety?

In teaching the young man continence he is also taught to regard woman in the most sacred sense; that the expression of any sexual irritation is extremely improper; that every courtesy and safeguard should be shown her in deference to her sex. Courtship is but the preparation for sexual selection, and the supreme ideal for the young man to hold is that sexual gratification comes only as the reward of requited love. To regard the favor of a woman in any other light is debasing and subjubates her to slavery—the only slavery woman knows.

How different, then, for young people to live up to the ideal when each is uncertain of the meaning and intentions of the other. And, add to this situation the also reprehensible practice, which women boldly foster, when they dress in such a manner as to emphasize the sex sphere. When every fold of the dress, color and texture is utilized to accentuate the form and sex zones. The young man with his ideals of chivalry and the buoyancy of youth sallies forth to enjoy the evening (in the refining company of the ladies) with mingled feelings of pleasure and expectations. His esthetic sense is charmed, for has not his fair lady employed all the embellishments of the feminine touch to charm and captivate her company? All might be well (and herein lies the real incentive of the dress, though apparently unconsciously affected), but for the instinct nature planted in the breast of every normal man, for the perpetuation of the race, which responds normally to the stimulus thus afforded, and his emotions are at once in conflict with the artificial decree of society. He naturally argues why this restraint to his inborn promptings, and nothing but the strength of another natural instinct—the moral character—will save him from asserting his role of victor.

When we teach the boy that continence is consistent with all physiological demands, we council him in the avoidance of constant sexual stimulus, for, indeed, continence is harmful in face of this irritant. Most of the nervousness and some of the insanity of the country is due directly or indirectly to ungratified sexual desire. Most often this connection is not recognized, owing to the false ideals in society, and the wrong interpretation is placed on otherwise misunderstood feelings.

What is the trouble? Is it the inherent weakness of woman, or the lust of man, or both? Whatever the answer may be, the voice of Eugenics speaks in clarion tones against the suggestiveness in modern dress and customs, and points out the necessity of moral standards for both man and woman. While reproduction is the call of nature, its aim is best attained by conservation of the sexual force, and this can only be done by building higher the fences of moral restraint.

---

Anti-typhoid serum is now manufactured in the laboratory of the Health Department of Texas and furnished gratuitously to the indigent.

MARVIN L. GRAVES, M. D.,
President Texas State Medical Association.

Dr. Graves was born in Bosque county, Texas, in 1867. Received common school education; M. A. degree, Southwestern University, Georgetown, Texas; M. D., Bellevue Medical College, 1891; Post-Graduate course in New York and in Berlin; in 1899 was Superintendent of the Southwestern Texas Insane Asylum at San Antonio; resigned to accept position of Professor of Medicine in Medical Department of the University of Texas. Dr. Graves is also Lecturer on Mental and Nervous Diseases; member county, State and National medical societies. Mrs. Graves is a daughter of the late Dr. H. C. Ghent.

## Figures and Tables

**Figure f1:**